# Novel *RS1* mutations associated with X-linked juvenile retinoschisis

**DOI:** 10.3892/ijmm.2012.882

**Published:** 2012-01-10

**Authors:** JUNHUI YI, SHIQIANG LI, XIAOYUN JIA, XUESHAN XIAO, PANFENG WANG, XIANGMING GUO, QINGJIONG ZHANG

**Affiliations:** 1 Department of Ophthalmology, The Third Xiangya Hospital, Central-South University, Changsha 410013; 2 State Key Laboratory of Ophthalmology, Zhongshan Ophthalmic Center, Sun Yat-Sen University, Guangzhou 510060, P.R. China

**Keywords:** mutations, *RS1* gene, retinoschisis

## Abstract

To identify mutations in the retinoschisin (*RS1*) gene in families with X-linked retinoschisis (XLRS). Twenty families with XLRS were enrolled in this study. All six coding exons and adjacent intronic regions of *RS1* were amplified by polymerase chain reaction (PCR). The nucleotide sequences of the amplicons were determined by Sanger sequencing. Ten hemizygous mutations in *RS1* were detected in patients from 14 of the 20 families. Four of the ten mutations were novel, including c:176G>A (p:Cys59Tyr) in exon 3, c:531T>G (p:Tyr177X), c:607C>G (p:Pro203Ala) and c:668G>A (p:Cys223Tyr) in exon 6. These four novel mutations were not present in 176 normal individuals. The remaining six were recurrent mutations, including c:214G>A (p:Glu72Lys), c:304C>T (p:Arg102Trp), c:436G>A (p:Glu146Lys), c:544C>T (p:Arg182Cys), c:599G>A (p:Arg200His) and c:644A>T (p:Glu215Val). Our study expanded the mutation spectrum of *RS1* and enriches our understanding of the molecular basis of XLRS.

## Introduction

X-linked retinoschisis (XLRS, MIM 312700) is a hereditary retinal disease characterized by a splitting of the neurosensory retina, with a prevalence of 1:5,000 to 1:25,000 males worldwide (1). Typical fundus changes include radiating cysteic maculopathy in most cases and peripheral retinoschisis in half of the cases (2). However, the disease has a high degree of phenotypic variability (3–6), in which genetic testing is of value in confirming the diagnosis (4).

XLRS accounts for most congenital retinoschisis (2,7) and is due to mutations in the retinoschisin gene (*RS1*, OMIM 312700) localized on Xp22.13 (8,9). The encoded protein, retinoschisin, is secreted from photoreceptors and bipolar cells as a functional homo-octameric complex that is thought to play a role in cellular adhesion and cell-to-cell interaction (10).

Gene transference to mouse models of X-linked juvenile retinoschisis, which suggest gene replacement may be a possible future therapy for patients (11–13). Genetic diagnosis is the basis for gene transference in the future. Therefore, we have to fully understand the molecular basis of XLRS. To date, more than 160 different *RS1* mutations have been identified in patients with XLRS (http://www.dmd.nl/rs), including small intragenic deletions, nonsense and missense mutations, frame shift insertions and deletions, and splice site mutations. However, there are still some *RS1* mutations that remain unknown.

In this study, we analyzed the coding exons and the adjacent regions of *RS1* in patients from 20 unrelated Chinese families with XLRS. Ten hemizygous mutations, including 4 novel mutations, were detected in 14 families.

## Subjects and methods

Probands with XLRS from 20 unrelated families were enrolled in this study. Written informed consent was obtained from the participating individuals or their guardians prior to the collection of clinical data and genomic samples. This study was approved by the Internal Review Board of the Zhongshan Ophthalmic Center.

### Mutation detection

Genomic DNA was prepared from venous leukocytes. Six pairs of primers (Table I) were used to amplify the six coding exons and the adjacent intronic sequence of *RS1* (NCBI human genome build 37.2, NG_008659.1 for genomic DNA, NM_000330.3 for mRNA, and NP_000321.1 for protein). Touchdown polymerase chain reaction (PCR) was performed with decreasing 0.5°C per cycle from 64°C for the first 15 cycles then down to 57°C (the annealing temperature) for the remaining 21 cycles. GC buffer was used. DNA sequences of the amplicons were identified with ABI BigDye Terminator cycle sequencing kit version 3.1 (Applied Biosystems, Foster City, CA) on an ABI 3130 Genetic Analyzer (Applied Biosystems). Sequencing results and consensus sequences from the NCBI human genome database were compared by using the SeqMan II program of the Lasergene package (DNA Star, Inc., Madison, WI) and then aligned to identify variations. Each variation was confirmed by bidirectional sequencing. Mutation description followed the recommendation of the Human Genomic Variation Society (HGVS). Variations detected in patients were further evaluated in controls by sequencing 176 normal individuals.

The Sorting Intolerant From Tolerant (SIFT) program and the Polymorphism Phenotyping (PolyPhen-2) were used to predict whether an amino acid substitution was likely to affect the protein function (14,15).

## Results

### Mutation analysis

**Figure 1:**
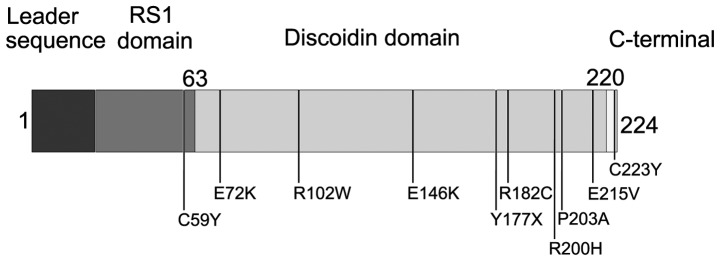
Sequence chromatography. Four novel sequence changes detected in the probands with RS are shown (left column) compared with corresponding normal sequences (right column).

**Figure 2:**
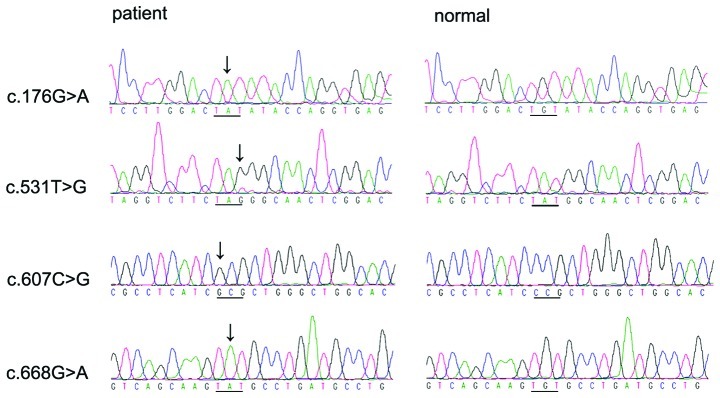
Protein sequence alignment of six RS1 orthologs. The regions with the novel p.C59Y and p.P203A mutations are highly conserved, C223Y is comparatively conserved.

Ten hemizygous mutations in *RS1* were detected in patients from 14 of the 20 families with retinoschisis (Table II and Fig. 1), including c:176G>A (p:Cys59Tyr) in exon 3, c:214G>A (p:Glu72Lys) and c:304C>T (p:Arg102Trp) in exon 4, c:436G>A (p:Glu146Lys) in exon 5, c.531T>G (p:Tyr177X), c:544C>T (p:Arg182Cys), c:599G>A (p:Arg200His), c:607C>G (p:Pro203Ala), c:644A>T (p:Glu215Val) and c:668G>A (p:Cys223Tyr) in exon 6. Of the 10, the c:176G>A, c:531T>G, c:607C>G and c:668G>A were novel. These novel mutations occurred in highly conserved regions (Fig. 2) and were predicted to be pathogenic (Table II). They were absent in 176 normal individuals.

All 10 probands with hemizygous *RS1* mutations (the clinical data of 4 probands were not available) had clinical symptoms and signs of retinoschisis (Table III). The four probands with novel mutations showed macular and peripheral retinoschisis.

## Discussion

**Figure 3:**
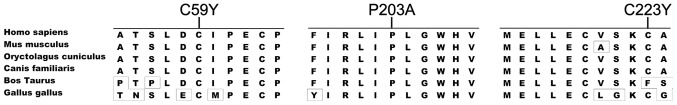
Distribution of the mutations detected a linear diagram of RS1 showing the organization of retinoschisin into domains and segments.

In this study, ten different hemizygous mutations in *RS1* were identified in 14 families with XLRS. These mutations are predicted to be pathogenic. All patients with mutations demonstrated typical signs of XLRS. The ten mutations affected different domains of retinoschisin, including the RS1 domain (1 mutation), discoidin domain (8 mutations) and C-terminal segment (1 mutation). These mutations were not randomly distributed over the gene (Fig. 3) because 80% of mutations were clustered in the discoidin domain (16). The two novel mutations, Tyr177X and Pro203Ala in the discoidin domain, may cause a shorter retinoschisin form or protein misfolding (13). The cysteine mutations in the *RS1* domain (Cys59Tyr) and C-terminal segment (Cys223Tyr) may cause failure of the discoidin domain to assemble into a normal multisubunit complex (17,18).

Most of *RS1* mutation loci were hot mutation spots, while the Cys59, Glu72, Arg102, Glu146, Arg182, Arg200, Pro203, Glu215 and Cys223 could be substituted by 1–2 other kinds of amino acids and be reported more frequently (19–30). However, the mutations in the present study also differed from those reported previously. The *RS1* mutations accounts for 70% of the Chinese retinoschisis (14/20) cases in our study. The Cys59Tyr, Tyr177X, Pro203Ala, Glu215Val and Cys223Tyr mutations only are present in the Chinese population (31), and the Cys59Tyr mutation was more common (10% frequency in our retinoschisis cases). The Glu72Lys mutation is the most common among Chinese (15%) as well as other populations (19,32), while another very common mutation, Pro192Ser (33), which was reported from people of different ethnic backgrounds was not found. We do not know whether the spectrum and frequency of *RS1* gene in the Chinese is different from others. Our study contributes to the current state of knowledge.

In summary, we identified ten mutations in 14 of 20 families with XLRS. Our results expand the mutation spectrum of *RS1* that might enrich our understanding of the molecular basis of XLRS in the Chinese population.

## Figures and Tables

**Table I:** Primers used for the amplification and sequencing of RS1.

Exon	Direction	Primer sequence (5′-3′)	Size of amplified fragment (bp)	Annealing temperature (°C)
1	F	GGTTAACTTGATGGGGCTCA	374	57
	R	AACTGGAAAGCCATCCACAC		
2	F	TCTATTTCACTTTTCCATGTAACGA	243	57
	R	ACCATGCCCAGCCAAAATA		
3	F	GACGATGCATAAGGACTGAGTG	296	57
	R	AGCGTTCAGGGGGTTAATTC		
4	F	GCAAAGCAGATGGGTTTGTT	359	57
	R	CCACCACGCCAGTTAATTTT		
5	F	CAGGGGGCTCTTTGGATG	389	57
	R	ACAGAGGGCAGTGACAGGAG		
6	F	CACCCGCAAACTGCTTTAAC	384	57
	R	TGCGAAATATAGCCCTGTCC		

GC buffer was used in all amplifications. F, indicates the forward sequence; R, indicates the reverse sequence.

**Table II:** The mutations of the *RS1* gene in XLRS.

				Computational prediction			Frequency			
							
Exon	Patient ID	Nucleotide change	Amino acid change	Blosum62	PolyPhen	SIFT	Patients	Controls	Note	Ref
3	QT42, QT335	c:176G>A	p:Cys59Tyr	9→-2	0.996	0	2/20	0/176	Novel	
4	QT221, QT232, QT653	c:214G>A	p:Glu72Lys	5→1	0.998	0	3/20		Reported	(19)
4	MD15	c:304C>T	p:Arg102Trp	5→-3	1	0	1/20		Reported	(20)
5	RP6	c:436G>A	p:Glu146Lys	5→1	0.961	0.17	1/20		Reported	(21)
6	MD30	c:531T>G	p:Tyr177X				1/20	0/176	Novel	
6	QT417, QT212	c:544C>T	p:Arg182Cys	5→-3	1	0.01	2/20		Reported	(22)
6	QT848	c:599G>A	p:Arg200His	5→0	1	0	1/20		Reported	(23)
6	QT911	c:607C>G	p:Pro203Ala	7→-1	1	0.13	1/20	0/176	Novel	
6	QT219	c:644A>T	p:Glu215Val	5→-3	1	0	1/20		Reported	(31)
6	QT758	c:668G>A	p:Cys223Tyr	9→-2	0.996	0.01	1/20	0/176	Novel	

All mutations are hemizygous.

**Table III:** Clinical information on individuals with *RS1* variations.

	Mutations		Age (years)			BCVA							
										
Patient ID	Nucleotide	Protein	Exam	Onset	Family history	OD	OS	Macular change	Peripheral change	Retinal hole	Strabismus	OCT	ERG(b/a)
QT042	176G>A	Cys59Tyr	N/A	N/A	No	N/A	N/A	N/A	N/A	N/A	N/A	N/A	N/A
QT335	176G>A	Cys59Tyr	11	6	No	0.4	0.2	mRS	pRS	No	No	RS	N/A
QT221	214G>A	Glu72Lys	19	EC	Yes	0.1	0.2	mRS	PD	No	No	N/A	N/A
QT232	214G>A	Glu72Lys	18	8	No	0.4	0.2	mRS	Degenenation	No	No	N/A	N/A
QT653	214G>A	Glu72Lys	5	3	No	0.3	0.7	mRS	pRS	Yes	No	N/A	Reduced
MD015	304C>T	Arg102Trp	N/A	7	No	0.2	0.3	PD, FRB	No	No	No	N/A	N/A
RP006	436G>A	Glu146Lys	5	4	No	FC	0.03	PD, FRB	No	No	No	N/A	Reduced
MD030	531T>G	Tyr177X	6	5	No	0.3	FC	mRS	pRS	No	Yes	N/A	Reduced
QT212	544C>T	Arg182Cys	N/A	N/A	N/A	N/A	N/A	N/A	N/A	N/A	N/A	N/A	N/A
QT417	544C>T	Arg182Cys	12	EC	No	0.3	0.03	No	pRS	Yes	No	N/A	N/A
QT848	599G>A	Arg200His	21	EC	No	0.6	0.4	mRS	No	No	No	N/A	Reduced
QT911	607C>G	Pro203Ala	22	EC	No	0.2	0.4	mRS	pRS	No	Yes	N/A	N/A
QT219	644A>T	Glu215Val	N/A	N/A	N/A	N/A	N/A	N/A	N/A	N/A	N/A	N/A	N/A
QT758	668G>A	Cys223Tyr	9	6	No	0.4	0.3	mRS	pRS	Yes	No	RS	N/A

BCVA, best-corrected visual acuity; mRS, macular retinoschisis; pRS, peripheral retinoschisis; RS retinoschisis; EC, early childhood; N/A, not available; PD, pigmental disorder; FRB, foveal reflex was blunted; FC, figure counting; ERG(b/a), the ratio of b wave amplitude to a wave amplitude.
